# Road polishing: High-resolution textures dataset of three mosaics made of different aggregates at different polishing stages

**DOI:** 10.1016/j.dib.2022.108594

**Published:** 2022-09-12

**Authors:** Malal Kane

**Affiliations:** Université Gustave Eiffel, Allée des Ponts et Chaussées, 44300 Bouguenais, France

**Keywords:** Road surface texture, Traffic polishing, Mosaics of aggregates, Type of aggregates, Texture evolution due to polishing

## Abstract

This database is composed of topographies of three mosaics of three different aggregates at three different polishing stages. Each topography is in two different formats: “sur” and “txt”. The “txt” format is composed of 15 parallel profiles separated by 0.5 mm. Each profile has a length of 76 mm. Every two points of the profile are separated by 0.01 mm. The three circular mosaics of 22.5 cm in diameter are made from three different aggregates: Limestone, Basalt, and Granite. They have been prepared by placing the 7.2-10 mm size fraction of the aggregates in a single layer as closely as possible, with their flattest faces lying on the bottom of a mold, and then filling the mold with a resin. The polishing process is made using the dedicated head of the Wehner-Schulze machine. The polishing head contains three rubber cones mounted on a rotary disc. It is the loaded and rolling action of these three cones on the pavement surface that produce the effect of polishing. To accelerate the polishing, a mix of 5% quartz powder in 95% water is sprinkled during the rotations of the cones. Texture has been captured using a laser scanning profilometer before, and after 90,000 and then 180,000 cycles of polishing. The measuring area is located inside the polished ring of the pavement.

**Specifications Table**

**Table:** 

Subject	Civil and Structural Engineering
Specific subject area	Road engineering
Type of data	The ".sur" files can be read by any mapping software (e.g. Mountainmap ® ...) and the ".txt" files can be opened directly by any software able to read ".txt" files.
How data were acquired	Texture has been captured using a laser scanning profilometer Stil Micromesure ®. This datalogger is equipped with the CHR high resolution optical sensor. It has a modular multi-axis structure (XYZ) and is thus equipped with motorized translation tables controlled from a PC. The resolution on the three axes (X, Y and Z) is 0.1 µm. An in-house developed software was used to separate the 3D images in ".sur" format into profiles in ".txt" format. This software allowed to correct the raw data by filling in the unmeasured points and the outliers.
Data format	Raw
Parameters for data collection	The data are ".sur" and ".txt" files. The ".txt" format is composed of 15 profiles separated by 0.5 mm. Each profile has a length of 76 mm. Every two points of the profile are separated by 0.01 mm.
Description of data collection	Texture has been captured using a laser scanning profilometer before, and after 90,000 and then 180,000 cycles of polishing. The measuring area is located inside the polished ring of the pavement. The topography was captured with 15 parallel profiles, 76 mm long, sampled every 0.01 mm, and separated by 0.5 mm intervals. The data are in the ".sur" and ".txt" format and are classified as follows: three directories, each representing a type of aggregate (Limestone, Granite, and Basalt). Each of these directories contains three other directories indicating the different levels of polishing (0, 90 000, and 180 000 polishing cycles). In each of these directories, there is a ".sur" file of the texture cartography, which is splitted in 15 profiles presented in ".txt" format.
Data source location	Institution: Université Gustave Eiffel City/Town/Region: 44 340 Bouguenais Country: France
Data accessibility	Kane, Malal (2022), “Road polishing: High-resolution textures dataset of three mosaics made of different aggregates at different polishing stages”, Mendeley Data, V1, doi:10.17632/j7sx7d786n.1 https://data.mendeley.com/datasets/j7sx7d786n/1
Related research article	Malal Kane, Michael Lim, Minh Tan Do, Vikki Edmondson, A new predictive skid resistance model (PSRM) for pavement evolution due to texture polishing by traffic, Construction and Building Materials, Volume 342, Part B, 2022, 128052, ISSN 0950-0618, https://doi.org/10.1016/j.conbuildmat.2022.128052.

## Value of the Data

These data are of paramount importance to the scientific community conducting research on pavement skid resistance. This is the first time that the community will have free access to data on pavement textures and their evolution when subjected to polishing by the Wehner Shulz machine (simulating road traffic). The study of these data can thus allow a better understanding of the effect of the type of aggregate and of the traffic on the skid resistance of road pavements.

To summarize,

• the gives access to high-resolution pavement texture (10 microns of resolution) made with different aggregates and at different polishing levels
• the effect of polishing on the evolution of the texture according to the type of aggregate can be analyzed with this database.

## 1 Data Description

The data are topographies captured from three mosaics of three different aggregates at three different polishing stages. The topographies have been captured using a laser scanning profilometer before, and after 90,000 and then 180,000 cycles of polishing. The measuring area is located inside the polished ring of the pavement. The topography was captured with 15 parallel profiles, 76 mm long, sampled every 0.01 mm, and separated by 0.5 mm intervals.

The data are in the ``.sur'' and ``.txt'' format and are classified as follows: three directories, each representing a type of aggregate (Limestone, Granite, and Basalt). Each of these directories contains three other directories indicating the different levels of polishing (0, 90 000, and 180 000 polishing cycles). In each of these directories, there is a ``.sur'' file of the texture cartography, which is splitted in 15 profiles presented in ``.txt'' format [2].

## 2 Experimental Design, Materials and Methods

**Fig. 1:**
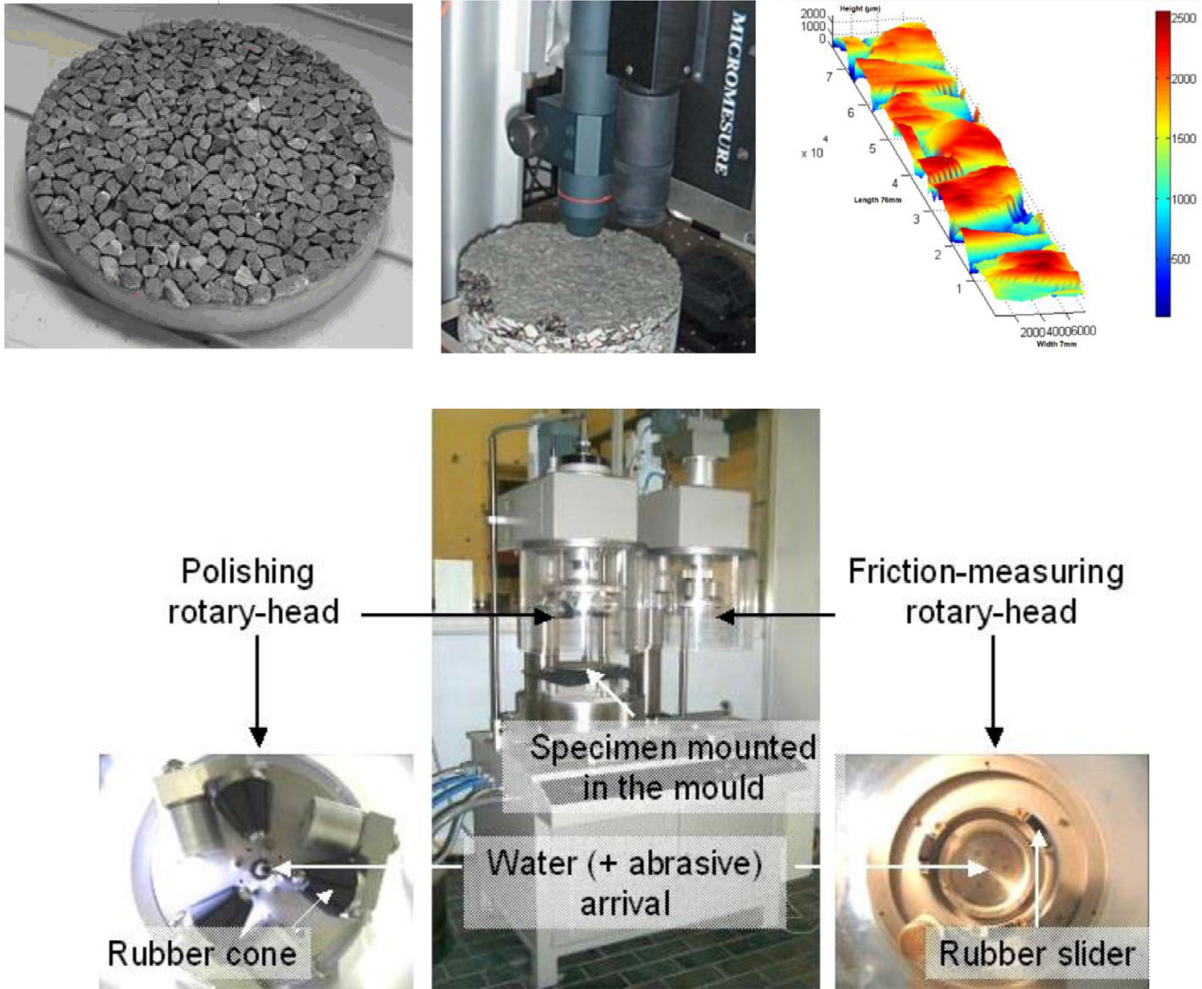
up left: Example of a pavement mosaic, up the middle: Profilometer, upright: Example of recorded topography, down: Wehner-Schulze machine. Modified from [1]

The topographies come from the capture of three mosaics of three different aggregates at three different polishing stages. The three circular mosaics of 22.5 cm in diameter are made from three different aggregates: Limestone, Basalt, and Granite. They have been prepared by placing the 7.2-10 mm size fraction of the aggregates in a single layer as closely as possible, with their flattest faces lying on the bottom of a mold, and then filling the mold with a resin. The polishing process is made using the dedicated head of the Wehner-Schulze machine. The polishing head contains three rubber cones mounted on a rotary disc. It is the loaded and rolling action of these three cones on the pavement surface that produce the effect of polishing. To accelerate the polishing, a mix of 5% quartz powder in 95% water is sprinkled during the rotations of the cones. Texture has been captured using a laser scanning profilometer before, and after 90,000 and then 180,000 cycles of polishing. The measuring area is located inside the polished ring of the pavement [See Fig. 1].

## Ethics Statement

Nothing to declare

## CRediT authorship contribution statement

**Malal Kane:** Methodology, Data curation, Writing – original draft, Investigation, Validation.

## Declaration of Competing Interest

The author declares that he has no known competing financial interests or personal relationships which have or could be perceived to have influenced the work reported in this article.

## Data Availability

Road polishing: High resoluted textures dataset of three mosaics made of different aggregates at different polishing stages (Original data) (Mendeley Data).
